# Maternal Programming of Sexual Behavior and Hypothalamic-Pituitary-Gonadal Function in the Female Rat

**DOI:** 10.1371/journal.pone.0002210

**Published:** 2008-05-21

**Authors:** Nicole Cameron, Adina Del Corpo, Josie Diorio, Kelli McAllister, Shakti Sharma, Michael J. Meaney

**Affiliations:** Sackler Program for Epigenetics & Psychobiology at McGill University and the Douglas Mental Health University Institute, McGill University, Montréal, Canada; The Rockefeller University, United States of America

## Abstract

Variations in parental care predict the age of puberty, sexual activity in adolescence and the age at first pregnancy in humans. These findings parallel descriptions of maternal effects on phenotypic variation in reproductive function in other species. Despite the prevalence of such reports, little is known about potential biological mechanisms and this especially true for effects on female reproductive development. We examined the hypothesis that parental care might alter hypothalamic-pituitary-ovarian function and thus reproductive function in the female offspring of rat mothers that vary pup licking/grooming (LG) over the first week postpartum. As adults, the female offspring of Low LG mothers showed 1) increased sexual receptivity; 2) increased plasma levels of luteinizing hormone (LH) and progesterone at proestrus; 3) an increased positive-feedback effect of estradiol on both plasma LH levels and gonadotropin releasing-hormone (GnRH) expression in the medial preoptic region; and 4) increased estrogen receptor α (ERα) expression in the anterioventral paraventricular nucleus, a system that regulates GnRH. The results of a cross-fostering study provide evidence for a direct effect of postnatal maternal care as well as a possible prenatal influence. Indeed, we found evidence for increased fetal testosterone levels at embryonic day 20 in the female fetuses of High compared to Low LG mothers. Finally, the female offspring of Low LG mothers showed accelerated puberty compared to those of High LG mothers. These data suggest maternal effects in the rat on the development of neuroendocrine systems that regulate female sexual behaviour. Together with studies revealing a maternal effect on the maternal behavior of the female offspring, these findings suggest that maternal care can program alternative reproductive phenotypes in the rat through regionally-specific effects on ERα expression.

## Introduction

Few genotype-phenotype relations would seem as potentially invariant as those between the genes of the sex chromosomes and the sexual differentiation of neuroendocrine function and behavior in mammals [1]–[4]. These effects are critical for reproduction. Nevertheless, many species reveal considerable *within-sex* variation in mating behaviors that reflect phenotypic plasticity in reproductive behavior [5]–[7]. In certain cases such phenotypic plasticity is stable over the lifespan and derived form parental effects [8], [9].

The neuroendocrine mechanisms underlying alternative reproductive phenotypes are generally unknown, especially for females where phenotypic variation has been relatively ignored [6]. Interestingly, perhaps the strongest evidence for phenotypic plasticity in females emerges from studies of reproductive development in humans [10] where there is evidence for parental effects on pubertal development and sexual behavior. Stressful environments that compromise parent-child interactions, especially those between the mother and daughter, advance the age of puberty and the onset of sexual behavior, increase the number of sexual partners in adolescence, result in a younger age at first pregnancy, a greater risk for unintended pregnancy and an increased number of sexually-transmitted infections [11]–[16]. These findings suggest that parent-child interactions in humans influence reproductive development in females. However, by necessity, relevant human studies are largely correlational; there is no evidence for a direct parental effect on the reproductive development of the female offspring. Moreover, there is little or no information on the possible biological mechanisms for phenotypic variation in reproductive function in humans or other species.

In the following studies we examined the hypothesis that variations in maternal care in early life in the rat influence hypothalamic-pitutitary-gonadal function and sexual behavior in the adult female offspring. There is considerable variation in specific forms of maternal behavior among lactating rats, notably in the frequency of pup licking/grooming [LG; 17]. These stable variations in maternal behavior influence gene expression and stress responses in the offspring [18]–[20]. In the current studies we found evidence for effects of such variations in maternal care on reproductive development in female offspring, including the timing of puberty.

## Results

### Sexual behavior

**Figure 1:**
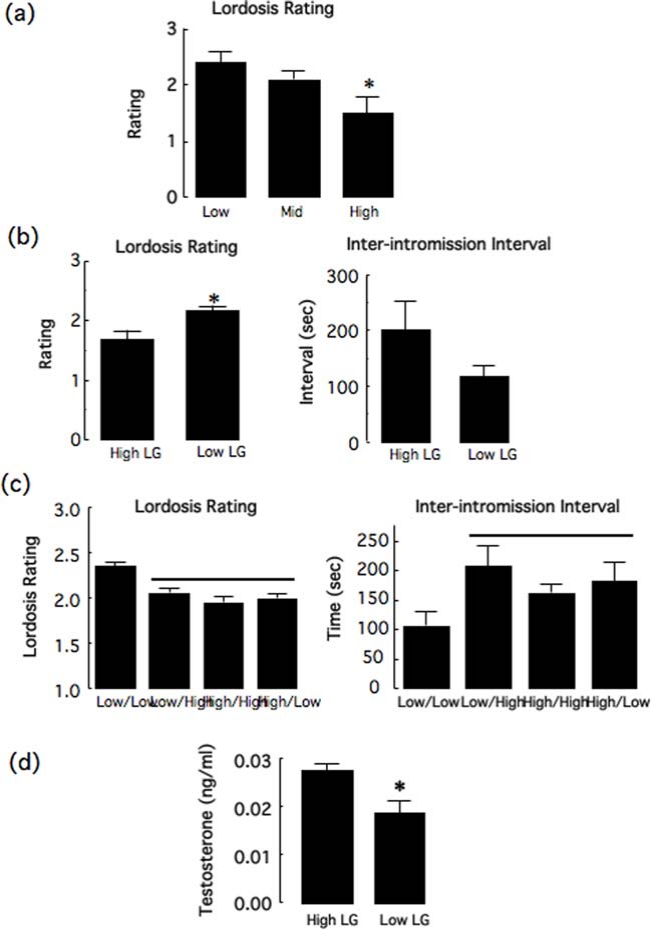
Mating behavior of the female offspring and the effect of cross-fostering. (a) Tested with a stud male in a small traditional testing arena, the mean (±SEM) lordosis rating in the adult female offspring of High LG mothers was lower compared to Low and Mid offspring (n = 10–12/group).* p<0.05. (b) Tested in a pacing chamber, the mean (±SEM) lordosis rating of adult female offspring of High LG mothers was lower compared to Low female offspring. * p<0.05. The inter-intromission interval showed no significant differences (n = 12/group). (c) Mean±SEM lordosis rating or inter-intromission interval in the adult female offspring of Low or High LG mothers fostered to and reared by Low (Low-Low; High-Low) or High (Low-High; High-High) tested with a stud male in the pacing chamber (n = 7–13/group). Groups lying underneath the line differ significantly (*p* <0.05) from the Low-Low group. (d) Mean±SEM testosterone level in amniotic fluid of embryonic day 20 female offspring of High or Low LG mothers (n = 20–25 pups from 12–14 litters/group; *p<.01).

Adult female offspring of High, Mid or Low LG mothers females were tested at the proestrus phase of the estrus cycle with a sexually experienced stimulus male in an arena (∼50×40×25 cm) typical of that used for testing female sexual receptivity in rodents. The sexual behavior of females for 15 intromissions [21] was videotaped and scored blindly. The results (Fig. 1a) revealed a significant effect [F(2,30) = 5.93; p<0.04] of maternal care on sexual receptivity. Post-hoc analysis the female offspring of Low LG mothers showed a significantly (p<.05) increased lordosis rating compared to those of High LG dams; the lordosis rating of the female offspring of the Mid LG mothers did not differ significantly from either of the other groups.

### Paced-mating study

We then tested a separate group of proestrus female offspring of High or Low LG mothers in a larger, multi-chamber apparatus, where the female, but not the male can move between the chambers. The female actively controls the pacing of the sexual interaction within this ‘pacing chamber’ [22]. The results (Fig. 1b) revealed a significant maternal effect on the lordosis rating [t(22) = 2.93; p<0.01] reflecting increased sexual receptivity in the adult female offspring of the Low LG mothers. There was a marginally significant maternal effect on intromission interval [t(21) = 1.51; p = 0.07] reflecting a trend for an increased intromission interval in the offspring of the High LG mothers (and see below). Also, the frequency of male-directed aggression (typically an upright boxing posture) was significantly higher in the female offspring of High LG mothers [t(22) = 2.12; p<0.03; data not shown]. The rejection of an attempted mount by a male occurred in 5/10 female offspring of High LG mothers and only 1/10 of the female offspring of Low LG dams. No differences were observed in the percentage of exit and the latency to return after a mount, intromission or ejaculation (data not shown).

### Cross-fostering study

We then examined the relation between postnatal maternal care and female sexual behavior. Pups (2–3/litter) were cross-fostered [20], [23] within 6 h of birth to mothers of the same (i.e., pups from High or Low LG mothers to other High or Low LG mothers, respectively) or alternative maternal phenotype (i.e., pups from High or Low LG mothers to Low or High LG mothers, respectively). Controls were similarly removed from their nest and the dam, and then returned to the biological mother (sham fostering).

Adult offspring were tested in the pacing chamber with a focus on the lordosis rating and the inter-intromission interval (Fig. 1c). Since there were no differences in the behavior of animals that were cross-fostered to mothers of the same biological origin (e.g., Low to Low) and the sham-fostered controls from dams of the same maternal phenotype (e.g., data from Low to Low offspring was pooled with that of the sham-fostered offspring of Low LG mothers, etc), we pooled these data, which then permitted a 2×2 ANOVA analysis of the effects of birth versus rearing mother. Statistical analysis of the lordosis rating revealed a significant effect of both birth mother [F(1,46) = 5.49; p<0.03] and rearing mother [F(1,46) = 4.23; p<0.05]. Post-hoc analysis revealed that the biological offspring of Low LG mothers fostered onto Low LG dams showed a significantly (p<0.05) higher lordosis rating than did animals in any other group including the biological offspring of Low LG mothers fostered onto High LG dams (Fig. 1c). These data reveal an effect of the rearing mother. In contrast, the lordosis rating of the biological offspring of High LG mothers was unaffected by the phenotype of the rearing mothers, thus accounting for the effect of birth mother. The same pattern of maternal effects was apparent for the inter-intromission interval data (Fig. 1c) revealed in the form of a significant interaction effect [F(1,46) = 6.13; p<0.03]. Post-hoc analysis revealed that the biological offspring of Low LG mothers fostered onto Low LG dams showed significantly (p<0.05) shorter inter-intromission intervals than did animals in any other group, including the biological offspring of Low LG mothers fostered onto High LG dams. Again, there was no cross-fostering effect on the biological offspring of the High LG mothers. These findings reveal an effect of both birth and rearing mother on the development of female sexual behavior in the rat.

### Amniotic testosterone (E-20)

The significant effect of ‘birth mother’ in the cross-fostering studies suggested a possible prenatal maternal influence. At embryonic day 20, the female fetuses of mothers previously characterized as High LG dams had a significantly higher level of testosterone in amniotic fluid than did females of mothers characterized as Low LG dams [*t* = 2.97, *p*<0.01]. There was no significant correlation between testosterone levels in the amniotic fluid of the female fetus and the number of males in a litter (r = −0.18, n.s.), the number of males in the uterine horn (r = −0.10, n.s.), or the number of males positioned above the target female in the horn (r = −0.06, n.s.). Moreover, we also found increased plasma levels of testosterone in the High compared to the Low LG mothers (Cameron, Del Corpo & Meaney, unpublished). These findings suggest that phenotypic variation in reproductive function in the female rat may include maternal influences apparent during both the pre- and post-natal periods.

### Hypothalamic-pituitary-gonadal (HPG) function at proestrus

**Figure 2:**
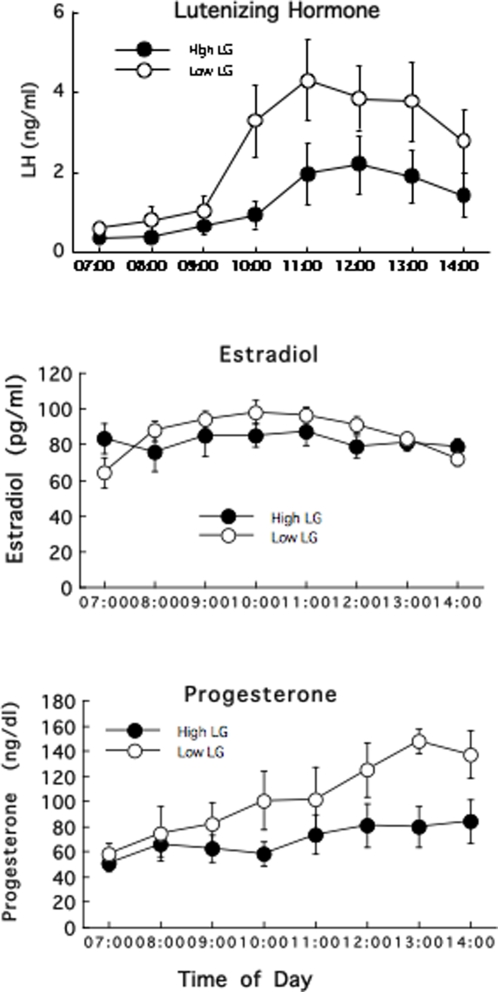
Hormonal levels during proestrus. Mean±SEM plasma levels of lutenizing hormone (LH), estradiol and progesterone in the adult offspring of High or Low LG mothers (n = 8–10/group) over the day of proestrus (lights off at 12:00).

The adult female offspring of High and Low LG mothers were sampled for plasma levels of luteinizing hormone (LH), estradiol and progesterone over the morning and into the afternoon of proestrus, a time that corresponds to the testing for female sexual behavior (Fig. 2). Plasma LH levels varied significantly over time [F(7,105) = 6.00; p<0.0001] and as a function of maternal care [F(1,15) = 4.70; p<0.05]. An area-under-the-curve analysis (AUC; performed using the Trapazoidal Rule) revealed significantly [t(16) = 2.45; p<0.02] higher plasma LH levels in the offspring of Low (2.76±0.55 ng/ml^−min^) compared to High LG mothers (1.28±0.37 ng/ml^−min^). There was no significant effect of maternal care on plasma estradiol levels (Fig. 2). In contrast, there was a significant effect of both time [F(7,98) = 5.04; p<0.0001] and maternal care [F(1,15) = 4.51; p<0.05) on plasma progesterone levels. The maternal effect was apparent in the later sampling period. Variations in plasma LH levels were associated with those in plasma progesterone. Across all animals, there was a significant correlation (r = 0.78; p<0.002; data not shown) between the area-under-the curve value for plasma LH and the mean level of plasma progesterone from 11:00 and 14:00h.

### LH/GnRH response to estrogen

**Figure 3:**
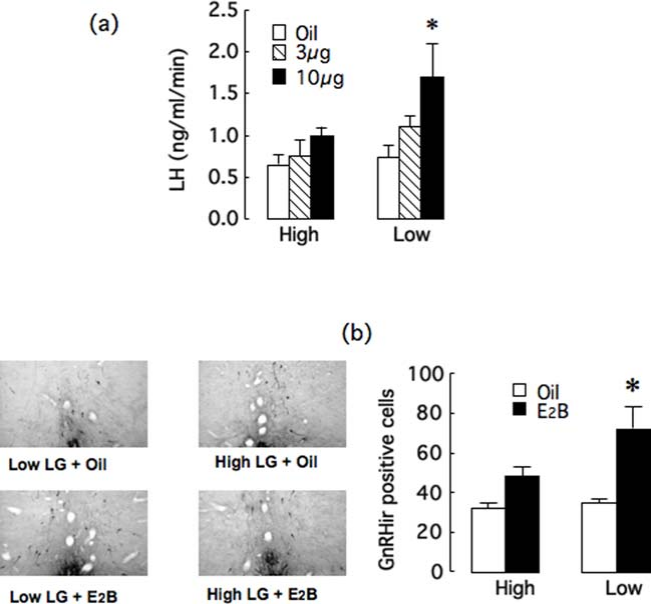
Estrogen effects on plasma LH and GnRH immunoreactive cells. (a) Mean±SEM plasma level of lutenizing hormone (LH) in ovariectomized adult female offspring of High or Low (n = 5–7/group) provided with low-level E2B replacement and then treated acutely with 0, 3 or 10 µg of E2B 24h prior to sampling. Adult female offspring of Low LG mothers treated with 10 µg of E2B showed a greater level of LH than all other groups (* p<0.05). (b) Photomicrograph of representative GnRH immunolabeling in the medial preoptic area of High or Low provided with low-level E2B replacement and then treated acutely with oil or 10 µg of E2B 24h prior to tissue sampling. The right panel reveals that the mean±SEM total number of GnRH-labeled cells located in the preoptic region (anteroventral preoptic nucleus, the median and lateral preoptic nucleus, the medial preoptic area) as well as the diagonal band nucleus in the Low offspring treated with E2B was greater than all other groups (n = 5/group;* p<0.05 ).

While estradiol drives the LH pulse [24]–[28], the proestrus, adult female offspring of High and Low LG mothers differ in plasma LH, but not estradiol (Fig. 2). This finding suggests increased sensitivity to the effects of estradiol on LH release in the female offspring of Low LG mothers. We examined this hypothesis in adult females that were ovariectomized and replaced with low levels of estradiol benzoate (E2B). The animals were treated acutely with 0, 3, or 10 µg of E2B and 24 h later were sampled hourly (09:00 to 14:00). The results (Fig. 3a) revealed a significantly greater effect of E2B on plasma LH levels in the female offspring of the Low compared with High LG mothers. Thus, plasma LH levels (AUC values) varied significantly as a function of treatment [F(2,24) = 3.63; p<0.05] and maternal care [F(1,24) = 4.18; p<0.05). Post-hoc analysis revealed that plasma LH levels were significantly (p<0.05) higher in female offspring of Low LG mothers treated with the higher E2B dose that in any other group, including the female offspring of High LG dams treated with the same dose of E2B.

Pituitary LH release lies under the dominion of the estrogen-sensitive, hypothalamic gonadotrophin-releasing hormone (GnRH) system [24]. Thus, we examined the effect of E2B on GnRH expression in a separate group of females using the 10 µg dose that distinguished plasma LH levels in the offspring of High and Low LG mothers. Animals were sampled 24h later (13:00h). As previously reported [e.g., 29], GnRH-immunocytochemistry (Fig. 3b) revealed that the majority of GnRH positive neurons were located in the preoptic area, particularly in the anteroventral preoptic nucleus (AVP), the median preoptic nucleus (MPN), the medial preoptic area (MPOA), the lateral preoptic nucleus (LPO), and the diagonal band nucleus (DBN). Only a few labeled cells were found in the medium septum (MS) and were excluded from the analysis. A limitation of this approach was the fact that in the anteroventral periventricular nucleus (AVPVn) staining was too dense to allow counting of individual cells.

The total number of GnRH-positive cells in the AVP, MPN, MPOA, LPOA and DBN were summed and statistically analyzed (Fig. 3b). The ANOVA revealed significant effects of E2B treatment [F(1, 22) = 24.90; p<0.001] and maternal care [F(1, 22) = 7.47; p<0.02], and a significant interaction between maternal care and E2B treatment [F(1, 22) = 5.02; p<0.04]. Post-hoc analysis showed that a significantly (p<0.05) greater number of GnRH-positive cells were found in E2B-treated offspring of Low LG mothers compared to Low control and both High LG groups. The groups did not differ significantly in the absence of E2B treatment.

### AVPVn estrogen receptor alpha (ERα) expression

**Figure 4:**
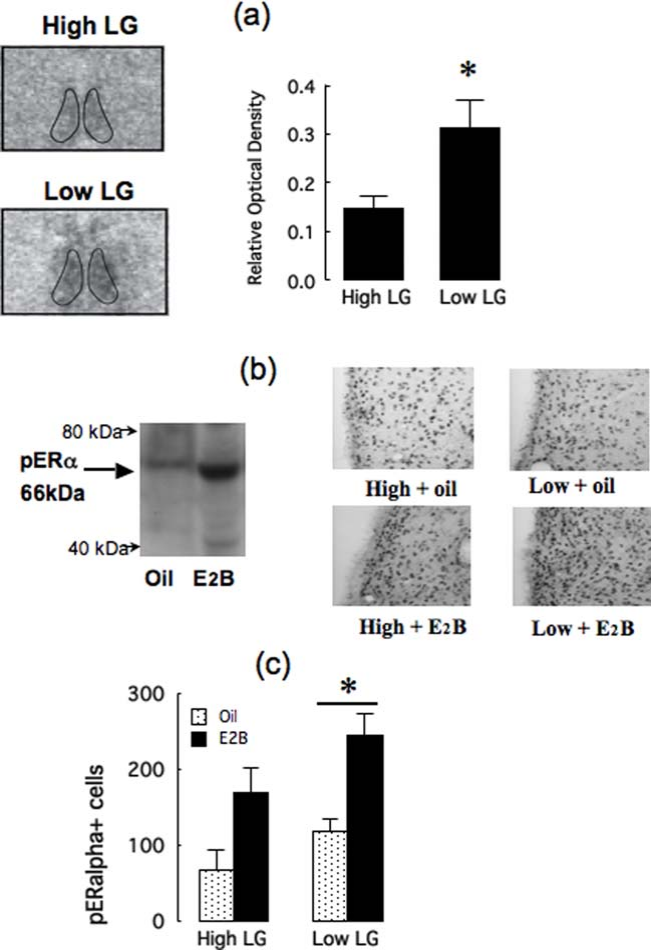
PhosphoERα mRNA and immunoreactivity in intact and estrogen-treated females. (a) Left panel shows representative autoradiograms of ERα mRNA expression in the anteroventral paraventricular nucleus of the hypothalamus (AVPVn; outlined region) in the adult female offspring of High or Low LG mothers. The right panel shows the mean±SEM for the quantification (relative optical density) of ERα mRNA in the AVPVn from the adult offspring of High or Low LG mothers (n = 5/group). * p<0.05. (b) Representative western blot depicting phosphoERα immunoreactivity at the expected 66kDa band in ovariectomized (O) treated with oil or 10 µg E2B, 90 min prior to tissue sampling. The Western blots revealed a limited banding pattern and the expected increase in phosphoERα−labelling. The right panels depict representative phosphoERα−labelling at the level of the AVPVn in ovariectomized adult female offspring of High or Low (n = 5–6/group) provided with low-level E2B replacement and then treated acutely with 10 µg of E2B or oil, 90 min prior to tissue sampling. (c) Mean±SEM number of phosphoERα−labeled cells in the AVPVn of the animals described above. Statistical analysis revealed a significant (p<0.05) overall effect of maternal care such that the number of phosphoERα−labeled cells was increased in the offspring of Low LG mothers in both treatment conditions.

The regulation of GnRH activity through estrogen receptor α (ERα) occurs at sites remote from the GnRH neurons [24], [25], [28]. The AVPVn shows dense ER**α** expression is critical for estrogen effects on the GnRH/LH surge [24], [27], [28]. Brain sections from intact adult female offspring of High or Low LG mothers were processed for *in situ* hybridization using ERα probes [30]. Autoradiographic film was scored (relative optical density) with reference to the Nissl-stained sections and the AVPVn was carefully outlined according to the rat brain atlas of Swanson (Plates 18/19) [31]. Statistical analysis of the data (Fig. 4a) summarized across these levels of the AVPVn (3 sections/animal) revealed a significant maternal effect [t(9) = 2.36; p<0.05] with greater ERα mRNA in the adult female offspring of Low LG mothers. A parallel study with separate group of adult female animals found no effect of maternal care on neuron density (i.e., quantification of the NeuN-labeled cells) or the number of cells (i.e., quantification of Nissl-stained sections) in the AVPVn (data not shown) suggesting that the increased ER**α** expression in the offspring of the Low LG mothers is not associated with differences in underlying morphology.

### PhoshoERα (pERα) expression

Estrogen binding increases phosphorylation of ERα primarily at serine-118 [32], which facilitates the recruitment of co-activators enhancing ER-mediated transcription. Thus, levels of ER**α** phosphorylation at serine-118 may provide a biologically relevant indication of ER**α** activation [32]. We examined pER**α** activation in the AVPVn using immunocytohistochemistry with an antibody specific to serine-118 pER**α**. The samples were obtained from animals prepared in the same manner as described above for GnRH expression and sacrificed at 60 min following E2B treatment. Preliminary studies using Western blotting (Fig 4b) revealed an expected increased in serine-118 pER**α** in E2B-treated ovariectomized animals compared with ovariectomy alone.

There was a significant E2B treatment effect [F(1,21) = 14.21; p<0.001] such that females that were ovariectomized and provided with low E2B replacement showed an increased number of pER**α**-labeled cells in the AVPVn following acute treatment with 10 µg of E2B (Fig. 4b,c). There was also a significant effect of maternal care [F(1,21) = 4.61; p<0.05]; across both treatment conditions (i.e., ovariectomy with low E2B replacement±acute E2B treatment) the number of pER**α**-labeled cells in the AVPVn was greater in the offspring of Low compared to High LG mothers. Ovariectomy with low E2B provided in implanted silastic capsules (see Methods and Materials) provides circulating estradiol levels in the range of the diestrus nadir [33], [34]; the 10 µg bolus injection produces elevated estradiol levels comparable to proestrus. Thus, there is increased pER**α**-labeling in the AVPVn in the female offspring of Low LG mothers across both low and high doses of E2B. This finding is consistent with that of an increased level of ER**α** expression in the AVPVn.

### Sexual behavior in E2B+progesterone-treated females

**Figure 5:**
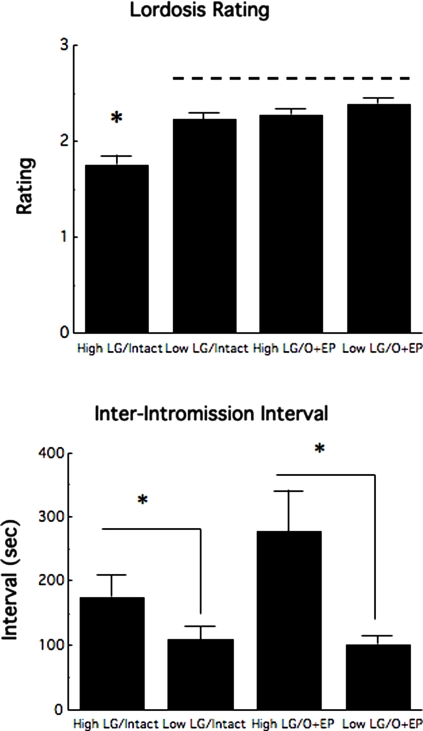
Effect of ovariectomy and steroid treatment in the sexual behavior of the female. Mean±SEM lordosis rating or inter-intromission interval in the adult female offspring of Low or High LG mothers tested with a stud male in the pacing chamber arena (n = 7–10/group) either intact (at proestrus) or following ovariectomy with E2B+progesterone replacement. For the lordosis rating data, groups lying underneath the line differ significantly (*p* <0.05) from the High LG/intact group such that the maternal effect is apparent among intact animals, but not ovariectomized animals with a common level of steroid replacement. For the inter-intromission interval data, the maternal effect is apparent (* *p* <0.05) among both intact and ovariectomy/steroid-primed conditions.

Sexual behavior in the female rat occurs as a function of estrogen and progesterone signals at proestrus. The results of the studies on HPG function suggest significant maternal effects on pituitary-ovarian activity at proestrus that might, in turn, underlie the differences in sexual behavior. We therefore compared the sexual behavior of the female offspring of High and Low LG mothers that were ovariectomized and replaced with both E2B (5 µg) and progesterone (500 µg) 4 and 44 hours prior to testing for female sexual behavior in the pacing chamber. Ovariectomized controls were not included in this study since these animals show little or no sexual receptivity. Instead, the behavior of the animals was compared to that of intact female offspring of High and Low LG mothers tested at proestrus. The results revealed that equalizing circulating levels of estrogen and progesterone completely eliminated the maternal effect on lordosis rating (Fig. 5). Statistical analysis revealed a significant maternal care×treatment interaction effect [F(1,30) = 3.82; p<0.05]. Post-hoc analysis revealed a significant (p<0.01) difference between the intact, female offspring of High and Low LG mothers. There was no such difference among the ovariectomized/steroid-replaced female offspring of High and Low LG mothers. Thus, the lordosis rating of the intact female offspring of High LG mothers was significantly (p<0.01) lower than that of any other group. Interestingly, the analysis of the inter-intromission interval data revealed a different pattern of results. Statistical analysis revealed only a significant maternal effect [F(1,30) = 6.05; p<0.02], with higher inter-intromission intervals in the female offspring of High compared with the Low LG mothers. The difference in the inter-intromission interval was apparent among both the ovariectomized/steroid-replaced and intact female offspring of High and Low LG mothers (Fig. 5). Thus, equating circulating levels of estrogen and progesterone in the female offspring of High and Low LG mothers was sufficient to eliminate the maternal effect on sexual receptivity, but not on the timing of mating.

### Puberty

**Figure 6:**
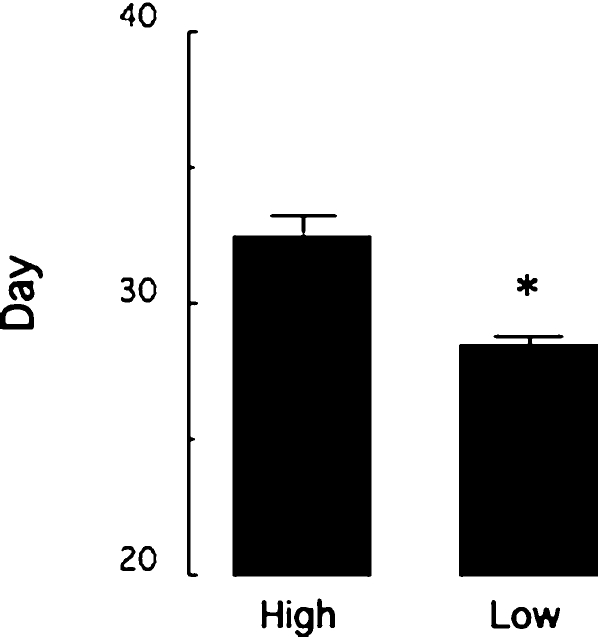
Onset of puberty. Mean±SEM day of vaginal opening in the female offspring of High (n = 16) and Low (n = 12) LG mothers revealing a maternal effect on the timing of pubertal development. * p<0.05

Vaginal opening is a reliable index of pubertal development in the female rat [35] and was observed daily between Day 21 and 35 in female offspring of High and Low LG mothers. There were no differences in the litter size at weaning or body weight at Day 32 as a function of maternal phenotype. Statistical analysis of the data revealed a significant maternal effect on the onset of puberty [t(27) = 2.35; p<0.05] with vaginal opening occurring earlier in the offspring of Low LG mothers (Fig. 6).

## Discussion

Variations in maternal care over the first week of life were associated with marked differences in the reproductive physiology and behavior of the adult female offspring.

The results of the reciprocal cross-fostering study reveal evidence for a postnatal, maternal effect on both lordosis and inter-intromission intervals (Fig. 1c). Analysis of the cross-fostering data revealed a statistically significant effect of ‘rearing mother’ on the measure of sexual receptivity (lordosis) as well as a significant interaction effect between ‘birth’ and ‘rearing’ mother for the inter-intromission interval data (Fig. 2). Both analyses therefore reveal evidence for an effect of rearing mother reflecting a direct influence of post-natal maternal care. Thus, the adult female offspring of Low LG mothers reared by High LG dams show significantly decreased sexual receptivity compared to littermates that were both born to and reared by a Low LG mothers. The same effect was apparent for the inter-intromission interval. Conversely, the sexual behavior of the biological offspring of High LG dams fostered onto Low LG mothers was indistinguishable from those born to and reared by High LG mothers. Accordingly, the statistical analysis revealed significant effects of both birth and rearing mothers. The post-natal effect of maternal care is consistent with previous studies showing that postnatal handling decreases sexual receptivity (i.e., lordosis ratings) in the adult female rat [36], [37]. Importantly, postnatal handling increases maternal LG in the rat [18], [38] and the effects of postnatal handling appear to be, in part at least, mediated by effects on maternal behavior [39], [40].

We previously observed a comparable pattern of cross-fostering effects on measures of hippocampal synaptic development and hippocampal-dependent learning [41], where likewise the offspring of Low, but not High LG mothers were affected by postnatal cross-fostering. This selective effect was related to the prenatal expression of NMDA receptor subunits linked to synaptogenesis, and suggested that under normal conditions the maternal effect on hippocampal development begins during prenatal life and continues into the postnatal period. Likewise, sexual differentiation in the rat occurs in response to both pre- and post-natal hormonal effects [1]–[3]. We found increased testosterone levels at embryonic day 20 in the female offspring of High LG mothers (Fig. 1d). Increased exposure to testosterone in late fetal development is associated with decreased positive-feedback effects of estradiol and a suppression of LH surges [42] as well as altered female sexual behavior [1]–[3 for reviews]. Each of these effects characterizes the adult female offspring of High LG mothers. Our findings suggest that such phenotypic variation emerges as a function of maternal effects over the course of the pre- and post-natal period that defines sexual differentiation in the rat.

The maternal effect on sexual receptivity appears to be functionally relevant [43]. The lordosis posture facilitates male intromission during mating [44]–[47]. Cameron et al. [43] found that during mating adult female offspring of High LG mothers received an increased number of mounts without intromissions (reflecting a decreased quality of lordosis), fewer ejaculations [43], and were less likely to be pregnant following mating. These findings suggest that the maternal effect on the sexual behavior of the female offspring is functionally important for reproduction.

Ovariectomy with E2B and progesterone priming completely eliminated group differences in lordosis, suggesting that the maternal effect on female sexual receptivity is mediated by differences in HPG activity at proestrus associated with differences in ERα expression in the AVPV. Among intact, cycling females, proestrus levels of LH and progesterone were significantly higher in offspring of Low compared with High LG mothers (Fig. 2). The preovulatory LH surge in the rat occurs in response to an estrogen “positive-feedback” influence on the GnRH neurons [24]–[32], [48], [49] that requires rising levels of estradiol coincident with a circadian input [24]–[26]. Estrogen acts at the AVPVn to stimulate GnRH gene expression [24]–[26], [38], [50]–[54]. Microimplants of antiestrogen into the AVPV also block both the estradiol-induced LH surge release and the phasic increase in GnRH gene expression seen several hours before the surge [27], [53], [54]. The AVPVn contains a dense population of ER-containing cells [55]–[58] that project directly to GnRH neurons with projections to the median eminence [24], [28], [59]. Neurons of the AVPVn are co-activated with GnRH neurons at the time of the LH surge [60]. ERα expression was increased in the AVPvn in the adult female offspring of Low compared with High LG mothers (Fig. 4) suggesting maternal influences on estrogen sensitivity of pathways that regulate pituitary LH secretion. Thus, increases in both GnRH labeling and plasma LH in ovariectomized females following E2B treatment were significantly greater in the female offspring of Low compared with High LG mothers (Fig. 3). These findings suggest that the differences in ERα expression in the AVPVn mediate, in part at least, the differences in HPG function and thus sexual receptivity in the female offspring of High and Low LG mothers.

Ovulation in the rat occurs even in the presence of a diminished LH surge [61], [62]. Moreover, studies [63] using a GnRH knockdown model suggest that ovulation and pregnancy can occur even with a greatly reduced number of GnRH-positive neurons. However, this outcome occurred under conditions where females co-habitated with males for several months. Interestingly, the heterozygote GNR23 mice, like the female of offspring of High LG mothers, show reduced GnRH expression and a suppressed LH surge. Moreover, the age of vaginal opening was delayed in the GNR23 +/− mice, similar to the findings with the offspring of High LG mothers. In our studies, females were mated under conditions that better approximate the mating conditions of the feral state. The results reveal evidence for decreased sexual receptivity [Fig. 1; 43] and pregnancy following mating [43] among the adult female offspring of High LG mothers. These findings suggest that the developmental plasticity of HPG function reflected in the maternal effects reported may regulate individual differences in sexual behavior. Indeed, among adult rats, variation in sexual receptivity is associated with variation in the preovulatory LH surge [64]. Our findings suggest that such variations can occur, in part at least, to maternal effects over the period of sexual differentiation.

In contrast to the AVPVn, ERα expression is increased in the adult female offspring of High compared with Low LG mothers [65], [66] in the medial preoptic area (MPOA) and is associated with differences in maternal behavior. Females reared by High compared with Low LG mothers show an enhanced estrogen-induced induction of oxytocin receptor binding in the MPOA and increased maternal LG during lactation [20], [66]. The maternal effects on ERα expression in the MPOA and pup LG in the female offspring are both completely reversed with postnatal cross-fostering, with no effect of birth mother. The differences in ERα expression in the MPOA are associated with differential methylation of a Stat5 binding site on the exon 1B promoter of the ERα gene [65], [66]. These findings suggest that maternal influences may program alternative reproductive tactics in the female offspring through tissue-specific stable, epigenetic effects on ERα expression.

There is considerable evidence for parental effects on reproductive development in the human female. Belsky et al. [11] suggested that environmental adversity is associated with a decreased quality of parental care that leads to an earlier onset of menarche and sexual activity in human females. Insecure attachment, child neglect and abuse are more prevalent in populations living under impoverished conditions [16], [67], [68] and there is strong evidence for familial influences, including mother-daughter relations [69], [70], on pubertal development and the onset of sexual activity [13], [15], [67], [69]–[74]. In contrast, familial cohesion and increased parental investment (e.g., time spent with children) are associated with a later onset of sexual maturation [13], [70], [75]. Importantly, early menarche predicts a younger age at first intercourse [15], [76]–[80], first pregnancy and birth of first child [79]–[81]. We found that variations in maternal care were associated with the age of puberty in the rat, suggesting that such effects may be common across a wide range of species.

These pervasive effects of parental care on reproductive development in the offspring may reflect a process whereby natural selection has shaped the offspring to respond to subtle variations in parental care as a forecast of the environmental conditions they will ultimately face following independence from the parent [7], [82]. The rationale for such ‘phenotypic plasticity’ is that under high-risk environmental conditions, when the probability of extended periods of growth and survival are low, the optimal strategy is maximize the number of offspring through accelerated mating, increasing the chances that at least some offspring will survive to reproductive maturity [14], [83], [84]. Moreover, since adverse environments are characterized by high, unavoidable risks and thus increased mortality, parental investment in offspring quality may be futile [83]. Such conditions favor a shift in reproductive investment towards quantity [10], [83]. In contrast, more propitious environmental conditions favor greater investment in individual offspring at the cost of mating, since offspring quality predicts successful competition for available for resources and reproductive fitness. Thus the quality of the prevailing environment defines parental investment, which in turn, is reflected in the mating and parental behaviour of the offspring. If true, then conditions that favor early sexual development in human females, should also diminish the parental care of the offspring; decreased parental investment in female offspring should results in a comparable pattern of parenting in daughters. In humans and other species there is indeed evidence that individual differences in parental care are transmitted across generations [20], [42], [85]–[88].

Variations in reproductive tactics among members of the same species [5], [6] suggest that evolutionary pressures produce phenotypic diversity rather than a single optimal strategy for males or females [5], [6], [9]. This seems only reasonable. Reproductive fitness is determined by the ability to survive to sexual maturity, reproduce and rear the offspring to reproductive age. Success reflects energy investments in reproductive processes, as well as in growth and survival [89]–[91]. Resources are limited; allocation for survival limits investment in reproduction, etc. The challenge is to establish the most effective investment strategy and the efficacy of potential solutions varies across environments. There is no optimal strategy-one phenotype does not fit all. We suggest that stable variations in reproductive life histories of female mammals can, in part, derive from parental influences during both fetal and postnatal development resulting in tissue-specific, epigenetic programming of ERα expression in brain regions that regulate maternal behavior (MPOA) and HPG function (AVPVn).

## Materials and Methods

### Animals

Animals were Long-Evans rats born in our colony and derived from breeders acquired from Charles River Canada (St. Constant, Quebec). Pregnant females were housed in a separate colony room maintained on a 10-h light, 14-h dark schedule with lights on at 0900 h unless otherwise stated. Food and water were provided *ad libitum*. The offspring were weaned on day 22 of life, housed in same-sex groups of 4 animals/cage until day 45 and then 2/cage until testing (90–120 days of age). The stage of the estrous cycle was determined by vaginal lavage between 08:30 and 09:30. To avoid intact litter effects, no more than 1–2 pups from any single litter were used in any of the studies. All testing and sample collection or processing was performed by individuals unaware of the treatment group of the animals. All procedures were approved by the McGill Committee on Animal Care.

### Maternal behavior

Maternal behavior [17]–[20], [88] was observed for five, 75-min observation periods per day for the first 6 days postpartum for the occurrence of pup LG scored every 3 min. The data were expressed as the percentage of observations in which pup LG occurred. The mean±SD of the frequency of pup LG was calculated for individual cohorts (n = 40–60/cohort) of lactating dams. High LG mothers were defined as dams that displayed a frequency of pup LG that was 1SD greater than that for the mean of the cohort; Low LG mothers were defined as dams for which the pup LG scores lay greater than 1SD below than of the cohort mean. There were no differences in total contact with pups [17].

### Cross-fostering

On the day of birth, pups were cross-fostered within 12 hrs after birth following previously published procedures [20], [66]. We exchanged 2 female pups/litter in litters of 8–11 pups and 3 female pups in litters larger than 11 pups. Cross-fostered pups were marked by tattooing the left hindleg (India ink); the siblings were tattooed on their right hind leg.

### Sexual behavior

Females were placed individually into the testing chamber (50×40×25 cm) between 11:30 and 14:30 h on the day of proestrus with a sexually-experienced male (300–350 g) for 15 intromissions. Behavior was scored for the occurrence and intensity (lordosis rating; LR) of lordosis, and the timing of each intromission (including ejaculations) received by the female. The lordosis quotient (LQ) was defined as the mounts accompanied by a lordosis response and the mean intensity of lordosis (lordosis rating, LR) was scored on a 3-point scale (0 for no lordosis; 3 for maximum dorsiflexion of the back) [93]. The inter-intermission-interval (III; sec between intromissions) was derived from the temporal data.

### Paced mating

Animals were previously exposed to the pacing chamber. Proestrus females were placed individually into the testing chamber (37.5×75×30 cm) [47], [48] with a ‘stud’ male for 45 min or a maximum of 15 intromissions. The pacing chamber [22] was divided into three sections by two barriers each with a hole (radius = 2.5 cm) sufficiently large to permit the passage of the female, but not the male. The male was placed into and confined to the central area of the chamber, while the female could move easily between the three sections. In addition to the behaviors described above, we scored the number of female exits from the central region and the latency to return to the central area with the male after receiving a mount, intromission or ejaculation.

### Pituitary-ovarian function over cycle

Animals were implanted with indwelling jugular vein catheters on diestrus day and sampled 24 hrs later, from 07:00 to 14:00 h on the day of proestrus (with lights on at 12:00 to 00:00h). Blood samples were collected, replaced with saline and placed on ice immediately after collection and subsequently centrifuged at 4°C at 2500 rpm for 12 min. Plasma was stored at −80°C until assay for LH, estradiol and progesterone using radioimmunoassays.

### Radioimmunoassays

Blood samples (500 µl) were collected, replaced with saline and samples were placed in tubes containing EDTA and aprotinin (Trasylol) on ice immediately after collection and subsequently centrifuged at 4°C at 2500 rpm for 12 min. Plasma was stored at −20°C until assay for LH, E and P using radioimmunoassays. Plasma LH level was assayed using a highly specific rabbit anti-serum, anti-native rat LH (ALPCO diagnostics # 29-R002) with I^125^ as tracer. The detection threshold of the assay is 0.075 ng/ml. The intra- and inter-assay coefficients of variation were 8.2% to 6.8% respectively. Plasma E level was determined using an anti-serum generated in rabbits by exposure to 6-keto-Estradiol-17-B-6-Oxime-BSA (MP Biomedicals # 07-138102). The detection threshold of the assay is 3.334 pg/ml. The intra- and inter-assay coefficients of variation were 4.7% to 9.1% respectively. Plasma P level was determined using a anti-progesterone generated against 11α-hydroxyprogesterone-11α-hemisuccinate in rabbits (MP Biomedicals #07-170102). The detection threshold of the assay is 0.1 ng/ml. The intra and inter-assay coefficients of variation were 3.6% to 6.7% respectively. Plasma Testosterone levels were determined using an anti-testosterone antibody generated against testosterone-19-carboxymethylether-BSA in rabbits (MP Biomedicals #07-189102). The detection threshold of the assay is 0.0075 ng/ml. The intra and inter-assay coefficients of variation were 4.6% to 7.5% respectively.

### LH response to estrogen

Adult female offspring of High or Low LG mothers were ovariectomized and implanted bilaterally with estrogen capsules (40 mm, 1 mg/ml E2B) that mimic the low circulating levels of estradiol typical of diestrus and maintain normal estrogen sensitivity [37], [38]. Animals were injected four days later with 0, 3 or 10 µg of E2B in sesame oil. The animals were sampled 24h later from the tail vein hourly from 09:00 to 14:00h (with lights on at 12:00 to 00:00h) two days after the injection and the samples were assayed for plasma LH levels.

### Caesarian-derived sampling

Caesarian sections were performed between 08:00 and 12:00 on timed-pregnant females bred in our colony. The females were previously characterized as High or Low LG mothers with a previous litter. Individual differences in the frequency of pup LG across litters are very stable [17], [34], such that pup LG scores across the first two litters, as in this study, are highly correlated (with r values of approximately 0.90). The uteri were quickly removed and the amniotic fluid from each fetus was sampled, after which the placenta were ruptured and the gender and the location within the uteri of the fetuses were determined. The samples were put on ice and soon after frozen at –20°C until assay for testosterone using radioimmunoassay. Amniotic level of testosterone was determined using radioimmunoassay (see below). This approach was based on evidence suggesting that steroid levels in amniotic fluid are in equilibrium with those in plasma [94], [95].

### Puberty assessment

Female offspring of High (n = 15) and Low (n = 11) LG mothers from multiple litters were examined daily from day 22 to 35 of life to determine the day of vaginal opening. The offspring were weighed on day 32 of life.

### Western blotting

Samples (75 ug protein) were mixed with an equal volume of 0.125M Tris base, 20% glycerol, 4% SDS, and 0.005% bromophenol blue and then denatured for 5 min at 100°C and separated on Novex 4–12% Tris-glycine polyacrylamide get electrophoresis (PAGE) precast gels (Helixx Technologies) with stained molecular markers (SeeBlue; Invitrogen) loaded for reference. Aliquots (10 µg) were loaded onto Novex pre-cast gels and subjected to electrophoresis for 3 h at 100V. Proteins were electrophoretically transferred onto nitrocellulose membranes overnight at 160 mA. Membranes were blocked for 1 h on a rotator at RT with 5% Carnation dried milk in TBS-T (Tris, NaCL, 0.1% Tween-20, pH 7.6), washed in TBS-T for 5 min and incubated at 4°C with anti-rabbit phospho-Era (SER-118) polyclonal antibody (concentration1:1000; Cell Signaling) diluted in TBS-T. Membranes were rinsed in 2×5 min TBS-T washes and incubated for 2 h at RT with anti-rabbit HRP (concentration 1∶1500) in TBS-T. Following 6×10 min washes in TBS-T, bands were visualized by enhanced chemiluminescence (ECL, Amersham) and exposed to Hyperfilm (Amersham) before being developed. The blots were stripped and re-probed with ß-actin monoclonal antibody (concentration1∶500) to verify loading accuracy. Optical density readings for phospho-ERα band (66 kDa) were determined using an image-analysis system (MCID-4, Imaging Research, St-Catherines, Ontario).

### Immunocytochemistry

Adult females were ovariectomized (with low E2B replacement as described above) and 14 days later received E2B (10 µg) or sesame oil (control) at 13:00h. Twenty-four h later, the animals were anesthetized with sodium pentobarbital (Somlethal, 120mg/kg) and perfused intracardially with phosphate buffer (PBS; pH. 7.2) followed by 4% paraformaldehyde. Brains were post-fixed in 4% paraformaldehyde, cryoprotected in 25% sucrose-PBS solution and frozen at −80°C. Thirty-µm coronal sections were prepared through the forebrain using freezing microtome. Sections were subsequently stored at –20°C in Watson's cryoprotectant solution [96] consisting of glycerol, ethylene glycol, distilled H2O, and phosphate-buffered saline (3∶3∶3∶1 volume ratio) until used for GnRH immunocytochemistry.

Free-floating sections were incubated in 0.5% hydrogen peroxide and 3% normal goat serum in PBS for 1.5 hr followed by 24 hr incubation with anti-GnRH antiserum (polyclonal antibody raised in rabbits, LH5, kindly provided by Dr. Robert Benoit, McGill University) diluted at 1∶150000 and a goat anti-rabbit secondary antibody. Visualization of GnRH-immonureactivity was performed using biotinylated IgG (1∶400; Vector Lab), Vectastain avidin-biotin horseradish peroxidase kit (Vector lab.) and 3,3′-diaminobenzidine as the chromogen. The phosphorylation of ERα at serine-118 was examined using immunocytohistochemistry as described above using a polyclonal antibody raised in rabbits (1∶800) specific to serine-118 pERα (#Ser118; polyclonal antibody; Cell Signaling Technology) with a goat anti-rabbit secondary antibody.

The number of GnRH-positive cells in every nuclei from the medium septum to the arcuate nucleus (∼30 sections/animal) was counted using every fifth coronal section. Fibers in the AVPVn were too densely packed to allow the counting of individually-labeled cells. The same procedure was used for quantification of pERα-positive cells, which included the AVPv (3 sections/animal). The quantification of positively-labeled cells over all regions was performed using the atlas of Swanson [53]. Statistical analysis was based on the mean number of positively-labeled cells calculated for each animal/region.

### Sexual behavior in ovariectomized, steroid-primed females

Animals were ovariectomized with low E2B replacement and 14 days later injected with E2B (10 µg/0.1 ml sesame oil) followed 44h later by progesterone (500 µg/0.1 ml sesame oil). Mating tests were conducted as described above (see Sexual Behavior) 4h after progesterone treatment.

### *In situ* Hybridization

ERα mRNA in situ hybridization was performed as previously described [30], [61] using S^35^ labeled cRNA probes generously provided by Dr. Serge Rivest (CHUL, Université de Laval, Québec, Canada). The cRNA probe was derived from a cDNA corresponding to the entire coding region of the ERα gene [30]. Coronal sections (16 µm) were thaw-mounted on poly-L-Lysine coated slides and stored at –80°C until processing. Slides were fixed 10 min. in paraformaldehyde (4% v/v), rinsed twice in phosphate buffered saline (PBS), and placed in acetic anhydride (0.25% v/v) in triethanolamine (0.1 M)/2×SSC (3 M sodium chloride; 0.3 M citric acid trisodium salt in H_2_0, pH 7.0 ) to reduce non-specific binding. The slides were washed in ethanol, chloroform (100 %), and partially re-hydrating ethanol washes and air-dried (RT). ^35^S-labelled cRNA probes were transcribed using a T3 MAXIscript kit (Ambion) and applied (100 µl) to sections, covered with a UV treated acetate sheet, and incubated for 18 h at 55°C. The slides were then washed in 2×SSC (RT) followed by a series of single washes: 30 min in Rnase (20 µg/ml, 37 °C), 15 min in 1xSSC (RT), 30 min in 0.5xSSC (50°C), 30 min in 0.1×SSC (65°C), and one brief wash in distilled water (dH_2_O) before dehydration. Slides were then exposed to autoradiography film (Hyperfilm-βmax, Amersham) for 4–6 days. Relative optical density (ROD) readings were determined from 3 sections/animal using a computer-assisted densitometry program (MCID Systems; Imaging Research, St. Catherines, Ontario, Canada).

### Statistical analyses

The results form studies with multiple groups were analyzed using one- or two-way analysis of variance (ANOVA) models. All post-hoc comparisons were performed using Tukey post-hoc tests. Analyses comparing results from two groups were performed using Students *t*-test.
